# Trends in Healthcare Utilization Among BIPOC and Non-BIPOC Older Adults: 1996–2016

**DOI:** 10.1093/geroni/igaf122.3348

**Published:** 2025-12-31

**Authors:** Yanjun Dong, Kun Wang, Victoria RIzzo

**Affiliations:** SUNY at Albany, Albany, New York, United States; University of Alabama at Birmingham, Birmingham, Alabama, United States; University at Albany, SUNY, Albany, New York, United States

## Abstract

Background Racial and ethnic disparities in healthcare utilization persist among older adults. Limited access to preventive, treatment, and long-term care services among BIPOC (Black, Indigenous, and People of Color ) older adults has significant implications for health outcomes and aging equity. This study examines how these disparities evolved from 1996 to 2016 and explores the interactions of race, income, and education with time in shaping healthcare access. Methods Data from the 1996–2016 Health and Retirement Study (HRS) (N = 42,233) were analyzed using mixed-effects logistic regression models. Healthcare utilization was categorized into preventive care, treatment care, and long-term care. Covariates included demographics, socioeconomic status, health conditions, and insurance coverage. Results Non-Hispanic Black and Hispanic older adults were less likely than non-Hispanic White older adults to use preventive (OR = 0.52 and OR = 0.59, p < 0.001) and treatment care (OR = 0.68 and OR = 0.69, p < 0.001). Disparities in preventive care utilization widened post-2008. Long-term care utilization increased for non-Hispanic Black older adults (OR = 1.03, p < 0.01), widening disparities, while Hispanic older adults’ long-term care use declined over time. Discussion Findings reveal persistent and widening disparities in preventive and treatment care, emphasizing systemic barriers faced by BIPOC older adults. The increasing reliance on long-term care among non-Hispanic Black older adults may reflect limited access to community-based alternatives. Targeted policies are needed to address inequities, improve healthcare access, and ensure equitable aging experiences.

